# The Pathogenic Effects of Moroccan Very Virulent Infectious Bursal Disease Virus on Lymphoid Organs: A Comparative Study in Conventional Broiler and Specific-Pathogen-Free Chickens

**DOI:** 10.3390/vetsci12040319

**Published:** 2025-04-01

**Authors:** Charifa Drissi Touzani, Imane Maaroufi, Ikhlass El Berbri, Fatima-Zohra Sikht, Ouafaa Fassi Fihri, Noursaid Tligui, Mohammed El Houadfi, Siham Fellahi

**Affiliations:** Pathology and Veterinary Public Health Department, Institut Agronomique et Vétérinaire HASSAN II, P.O. Box 6202, Rabat 10000, Morocco

**Keywords:** pathogenicity, vvIBDV, broiler, SPF chickens, bursa of Fabricius

## Abstract

Infectious bursal disease (IBD) is a major immunosuppressive disease affecting young chickens, and causes significant economic losses to the poultry industry. This work represents the first pathogenicity assessment of Moroccan very virulent IBD virus. Molecular characterization and sequence analysis of this isolate previously identified specific substitutions, including seven amino acid substitutions in segment A, and I472L and E688D in segment B, specific and unique to Moroccan vvIBDV strains. Two chicken lines, broiler and specific-pathogen-free (SPF) chickens, were inoculated via the occulonasal route with 0.2 mL of the 10^5^EID_50_ /mL viral solution of the IB19 vvIBDV strain at 29 days of age. Experimental monitoring was carried out for 10 days post-challenge (dpc). Clinical signs started on the second dpc, with peak severity observed between 3 and 6 dpc. The total mortality rate reached 10% in broilers (group G1) and 93% in SPF chickens (G3). Macroscopic lesions in G1 broilers included marked hypertrophy of the bursa of Fabricius (BF), followed by very pronounced atrophy, while macroscopic examinations of deceased SPF birds (G3) revealed very hemorrhagic BF with a black cherry appearance in 80% of dead birds. The mean Bursa/Body Index (BBI) of challenged broilers (G1) showed a decrease of 46% compared to the control group (G2), indicating bursal atrophy. Microscopic lesions in the BF consisted mainly of inflammation, with severe lymphoid depletion of the follicles in challenged G3 SPF birds. This in vivo study of Moroccan vvIBDV demonstrated a distinctive virulence profile, and confirmed its classification as a very virulent strain with substantial disease-causing potential. It is crucial to obtain comprehensive knowledge of the prevalence, emergence, pathogenicity, and control of Moroccan IBDV strains.

## Simple Summary

Infectious bursal disease (IBD) is a highly contagious and immunosuppressive disease affecting young chickens, causing significant economic impact on the poultry industry. This work represents the first pathogenicity assessment of Moroccan very virulent IBD virus. This in vivo study of Moroccan vvIBDV demonstrated a distinctive virulence profile, and confirmed its classification as a highly virulent strain with substantial disease-causing potential.

## 1. Introduction

Infectious bursal disease (IBD), also known as “*Gumboro* disease”, is an immunosuppressive disease affecting young chickens between 3 and 6 weeks of age, and causes high mortality in young chickens, compromised vaccination efficacy, and reduced egg production [1,2]. Recently, with the re-emergence of mutants and novel variant strains, IBDV has acquired remarkable importance, causing significant economic losses in the poultry industry [3].

Infectious bursal disease virus (IBDV) is a non-enveloped RNA virus belonging to the *Avibirnavirus* genus within the *Birnaviridae* family [4]. IBDVs are classified according to their pathotype, serotype, and genotype [5,6,7,8]. There are two main serotypes of IBD virus: serotype 1 and 2. Serotype 1 is specific to the Gallus Gallus species, and includes various pathotypes, such as classical IBDV, variant IBDV, attenuated IBDV, and very virulent IBDV (vvIBDV). In contrast, serotype 2 is apathogenic for chickens [9]. Regarding the genetic characterization of IBDV, Islam and co-authors suggested new genetic classification [8]. There are eight genogroups for segment A (A1–A8), among which A3 is specific to very virulent IBDV strains, and five genogroups for segment B (B1–B5), with B2 corresponding to vvIBDV strains. Bi-segmentation of the genomes of IBDV has generated up to forty-five reassortments or variants, emphasizing the complexity of the genetic classification of IBDV [8].

Oral transmission is considered a common way in which chickens become infected with IBDV, by picking at feed, water, or materials contaminated by IBDV. The IBDV initially reaches and replicates in macrophages and lymphoid cells within the lamina propria of the intestinal mucosa. The virus then invades the bloodstream, and targets and replicates within immature B lymphocytes in the bursa of Fabricius (BF) organ. This leads to necrosis and depletion, and finally results in immunosuppression [10].

The pathotype is assessed through the study of clinical signs, mortality, and macroscopic and microscopic lesions. The severity of clinical signs and lesions depends on the chicken breed, the pathogenicity of the IBDV strain, and the age at infection. Layer breeds, most notably the White Leghorn, are more susceptible to the disease [2]. Additionally, very virulent (vv) pathotypes of IBDV strains cause severe lesions in the BF, as well as marked lesions in other lymphoid organs, inducing a 50–100% mortality rate in SPF chickens [11]. Clinically, acute infection with vvIBDV is characterized by severe depression, ruffled feathers, and white watery diarrhea. Symptoms and mortality appear suddenly, usually at 3 dpc, peak, and then recede in 5 to 7 dpc [2,12,13]. However, the virulence of IBDV strains varies greatly among different genogroups [7].

A detailed molecular and phylogenetic characterization of the hypervariable region (HVR) of the VP2 protein from infectious bursal disease virus (IBDV) strains circulating in Morocco was performed [14]. The analysis revealed that the isolated strains belonged to the very virulent genotype, highlighting the potential for increased pathogenicity [14]. Four representative strains were then carefully selected for complete IBDV genomic sequencing [15]. The results showed the circulation of very virulent Moroccan IBDV strains clustered within the A3 genogroup, as proposed by Islam et al. [8]. Further analyses positioned the Moroccan strains within very virulent genogroup A3B2, revealing distinctive mutational sites unique to Moroccan isolates. This funding suggests possible local adaptations or specific evolutionary pressures [15].

The complex nature of IBDV virulence [16,17] makes its evaluation crucial for understanding the IBD virus’s epidemiology and informing effective prevention and control strategies. Moreover, comprehensive genomic analyses of Moroccan IBDV isolates have confirmed the circulation and propagation of a strain belonging to a very virulent genotype, associated with significant economic losses in the poultry industry and characterized by a unique genetic signature in the HVR of the VP2 gene of vvIBDV [15]. Despite these molecular characterizations of vvIBDV strains, their pathogenicity has not yet been investigated. The aims of this study is to evaluate the pathogenicity of the Moroccan vvIBDV strain in two chicken lines: broiler and SPF chickens.

## 2. Materials and Methods

### 2.1. Animals

Fifty Cobb broiler chicks were reared in poultry pens at the Avian Pathology Unit at the Institut Agronomique et Vétérinaire Hassan II (Rabat, Morocco), starting from one-day-old. At this time point, blood samples were collected from ten chicks to determine maternal antibody levels against IBDV. The remaining forty chicks were vaccinated against infectious bronchitis, Newcastle disease, and H9N2 influenza virus, following a standard vaccination schedule consistent with the field conditions (Table 1). Twenty-five SPF chicks were obtained from MCI (Mohammedia, Morocco) at 5 days old, and were divided into two groups, housed in separate pens at the Avian Pathology Unit (Rabat, Morocco). The birds had ad libitum access to feed and water.

### 2.2. Experimental Design

Experiment 1. The broiler chickens were divided into two groups. Group 1 (G1) (*n* = 30) was tagged and challenged at 29 days of age via oculo-nasal routes with 0.2 mL of Moroccan vvIBDV strain (1/chicken/Morocco/IB19/2017 (accession number.MK580160, MK580164) [15]; viral titer: 10^5^ EID_50_/mL). G1 was further divided into two subgroups: A (*n* = 10) and B (*n* = 20). The subgroup G1A was used to record clinical signs and mortality rates for 9 dpc. Subgroup G1B was used to evaluate the macroscopic and microscopic lesions and conduct morphometric measurements. Group 2 (G2) (*n* = 10) served as a non-challenged control group. Clinical signs and mortality were monitored daily.

On 1, 3, 5, 7, and 9 dpc, four chickens from G1B and one control from G2 were randomly selected and sacrificed (euthanized). The body weights and macroscopic lesions of the euthanized birds were recorded. Morphometric measurements (as described below) were carried out on the BF, spleen, and thymus. Each collected bursa was bisected; one portion was stored at −20 °C for molecular analyses, and the second half was placed in 10% neutral buffered formalin for histopathological examinations. Tissue samples from the spleen, caecal tonsil, liver, kidneys, and thymus were collected for histopathological examinations. Cloacal swabs were collected from G1A on 2, 4, 6, 8, and 9 dpc and used for viral load assessment. At 9 dpc, all the remaining chickens were humanely sacrificed, and their BF and other organs were collected.

Experiment 2. Twenty SPF chicks were divided into two groups: the challenged group G3 (*n* = 15) and the unchallenged group G4 (*n* = 5). In addition, five birds were bled before the start of the trial for confirmation of the absence of antibodies against IBDV using an IDEXX FlockChek ELISA Kit (IDEXX Corporation, Westbrook, ME, USA). At 29 days of age, the birds were individually tagged. Only G3 was challenged with 10^5^ EID_50_/mL of Moroccan vvIBDV strain via the oculo-nasal route (0.2 mL/dose), and G4 was kept as a negative control. The clinical signs and mortality were monitored daily throughout 10 dpc, and BF samples were collected and divided in half for molecular and histopathological investigations.

### 2.3. Clinical Signs

After the challenge, birds of each group were individually examined for signs of IBD, including from depression associated with prostration, ruffled feathers, and white liquid diarrhea.

### 2.4. Histopathological Changes

Histological sections were made and stained with haematoxylin-eosin, following conventional procedures. Microscopic lesions were examined in the BF, caecal tonsil, spleen, thymus, liver, and kidneys in broilers, while only BF lesions were assessed in SPF chickens. Lesion severity was scored for the BF, caecal tonsil, spleen, and thymus.

BF lesions were scored using the method of Muskett et al. [18], as described by Van den Berg et al. [19]:

0 = no damage; 1 = scattered lymphoid necrosis in a few follicles; 2 = moderate lymphoid depletion in most of the bursal follicles, or isolated follicles with severe depletion; 3 = severe lymphoid depletion in almost all of the bursal follicles, which appear pale and vacuolated, and heterophil infiltration; 4 = outlines of follicles remaining with few lymphocytes, glandular transformation, cysts, an increase in interfollicular tissue, and lymph plasmatic infiltration; 5 = complete loss of follicular architecture with fibroplasia.

### 2.5. Quantification of IBDV Using Quantitative RT-PCR

A preliminary qualitative real-time RT-PCR was performed to assess the cycle threshold (CT) of the samples. The sample with a 10^5^EID_50_/mL titer was then chosen as a standard for the quantitative real-time RT-PCR.

A standard curve was made by serial 10-fold dilutions of the standard sample, and the standard curve was plotted using Microsoft Excel.

RNA extraction: The virus preparation was carried out from the individually harvested and centrifuged BF. The obtained supernatant was used for viral RNA extraction.

Cloacal swabs and BF supernatants were processed using the MACHEREY-NAGEL viral RNA isolation kit (MACHEREY-NAGEL GmbH & Co. KG, Duren, Germany), following the manufacturer’s instructions.

Real time reverse-Transcription PCR: A one-step real-time RT-PCR assay was carried out using an ABI Prism 7500 (Applied Biosystems, ThermoFisher Scientific, Waltham, MA, USA). The reaction was conducted with the Bioline SensiFAST kit (Bioline, a Meridian Life Science Company, London, UK). The primers and probes were used as described by Tomas et al. [20]. The reaction mixtures and the thermal cycling conditions followed the protocol outlined by Drissi Touzani et al. [14].

## 3. Results

Before the challenge, the birds used in this study were tested for detection of IBD antibodies. The serological analyses of day-old broiler chicks showed heterogenicity in maternal antibodies, reaching a mean titer of 7831. However, the SPF chicks revealed no IBD antibodies in their sera.

### 3.1. Clinical Signs and Mortality

#### 3.1.1. Experiment 1

**Figure 1:**
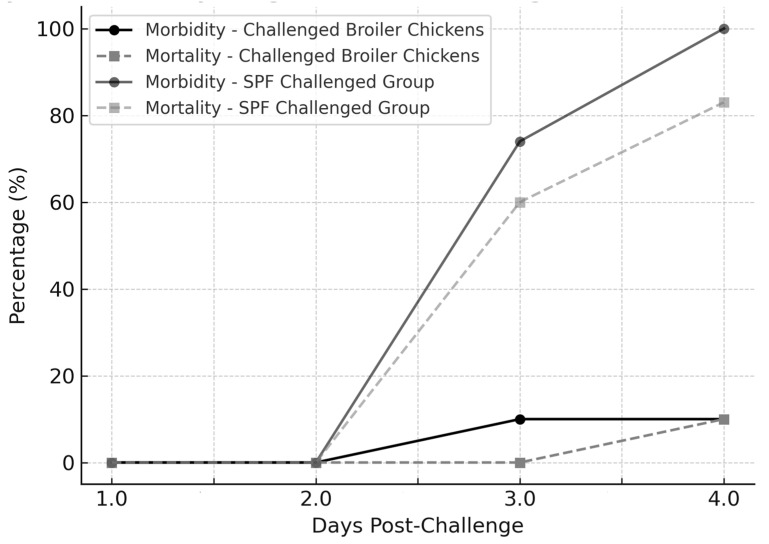
Morbidity and mortality progression in challenged broiler chickens and SPF chickens. This figure illustrates the morbidity and mortality rates in broiler and SPF chickens following vvIBDV infection.

Clinical signs of acute IBD (characterized by severe depression with prostration, ruffled feathers, and white watery diarrhea) were observed. They appeared suddenly at 3 dpc, peaked between 3 and 6 dpc, and decreased at 7 dpc. Mortality occurred at 4 dpc, and reached 10% (2/20). No mortality was recorded in negative control G2 (Figure 1).

#### 3.1.2. Experiment 2

Daily clinical observations of SPF chickens revealed the presence of prostration as early as 24 h post-challenge. At 48 h post-inoculation, some birds were reluctant to move, with ruffled feathers and white watery diarrhea. The clinical signs worsened at 3 and 4 dpc. Morbidity reached 74% and 100% (11/15–6/6) at 3 and 4 dpc, respectively. The mortality rate reached 60% (9/15) at 3 dpc and 83% (5/6) at 4 dpc, respectively, for the SPF challenged group (G3). However, no clinical signs were detected in birds of the negative control group (G4) (Figure 1).

*Morbidity—Challenged Broiler Chickens (●*, *black solid line)*: *Clinical signs appeared on Day 3 post-challenge and remained stable at 10%.*

*Mortality—Challenged Broiler Chickens (■*, *black dashed line): Mortality was first observed on Day 4 post-challenge, reaching 10%.*

*Morbidity—SPF Challenged Group (●*, *gray solid line): Morbidity increased sharply to 74% on Day 3 and 100% on Day 4, indicating a more severe disease progression.*

*Mortality—SPF Challenged Group (■*, *gray dashed line): Mortality reached 60% on Day 3 and 83% on Day 4, highlighting the higher susceptibility of SPF chickens to vvIBDV infection.*

### 3.2. Postmortem Examination

#### 3.2.1. Experiment 1

The macroscopic lesions observed on the BF were characterized by petechial hemorrhages in the bursal mucosa, as well as edema. These lesions peaked in severity at 4 dpc. Bursal atrophy was the predominant lesion from 7 to 9 dpc. After 5 dpc, most of the BF became small and yellowish. Splenic congestion with splenomegaly was observed from 3 dpc, peaked between 4 and 7 dpc, and decreased at 9 dpc. Thymic congestion was observed at 4 dpc.

#### 3.2.2. Experiment 2

**Figure 2:**
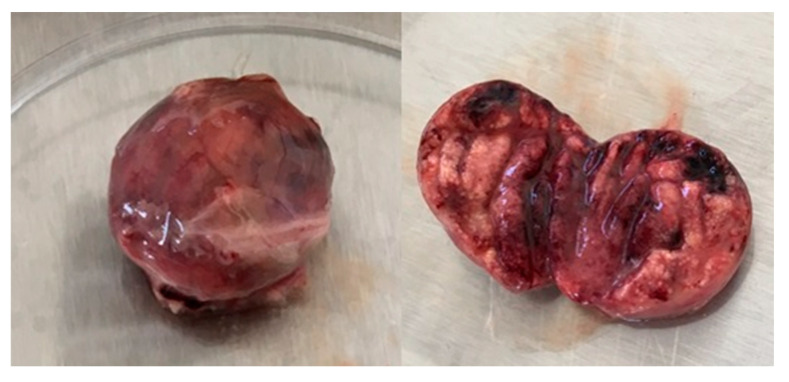
Bursa of Fabricius from SPF chickens challenged with the Moroccan vvIBDV strain at 3 days post-challenge (dpc). The left image shows a characteristic black cherry discoloration, indicative of severe hemorrhagic lesions. The right image presents a sagittal section of the bursa of Fabricius, revealing internal hemorrhage, edema, and swelling, which are hallmark lesions associated with vvIBDV infection.

A post-mortem examination was conducted on birds that died at 3 and 4 dpc (14/15), and on one remaining chicken sacrificed at 10 dpc. The frequent lesions observed in 80% of birds were severe and hemorrhagic BF and petechial hemorrhages on thigh and breast muscles (Figure 2). Other lesions were observed, such as splenomegaly and enlargement of the kidney, in 60% of birds. No gross lesions were detected in G4.

### 3.3. Morphometric Measurements

#### 3.3.1. Volume of Bursa of Fabricius

**Figure 3:**
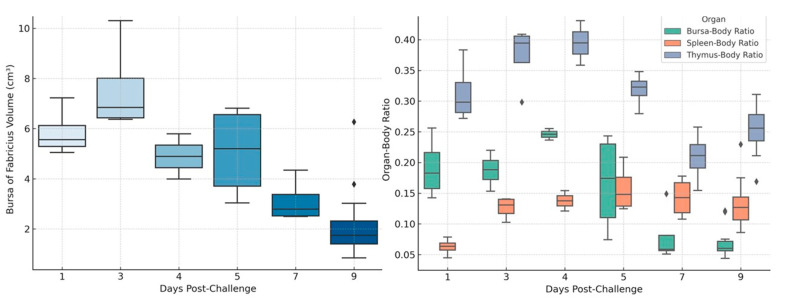
The bursa of Fabricius volume and organ–body ratio in challenged and non-challenged broiler chickens. **Left figure**: the bursa of Fabricius volume in challenged (G1) and non-challenged (G2) broiler chickens, illustrating the impact of vvIBDV infection on bursal atrophy over the post-challenge days. **Right figure**: the organ–body ratio (bursa–body ratio, spleen–body ratio, thymus–body ratio) in challenged (G1) and non-challenged (G2) broiler chickens, showing variations in lymphoid organ sizes following infection.

The volume of the BF decreased significantly in challenged broiler chickens (G1), ranging from approximately from 7 cm^3^ to 2.5 cm^3^. In contrast, the BF volume in control broiler chickens (G2) increased moderately (Figure 3 Left).

#### 3.3.2. Bursa–Body Ratio (BBR) and Bursa–Body Index (BBI)

Both the BBR and the BBI decreased notably in challenged broiler chickens (G1). In the first group, the BBR decreased from 0.225 to 0.075, whereas in non-challenged broiler chickens (G2), the BBR increased slightly from 0.200 to 0.250 (Figure 3 Right). Furthermore, the BBI revealed a distinct regression in challenged broiler chickens (G1) compared to non-challenged broiler chickens (G2), with values of 0.54 and 1, respectively.

#### 3.3.3. Spleen–Body Ratio (SBR)

The data revealed that challenged broiler chickens (G1) had a significant increase in SBR compared to the control group (G2), confirming the splenomegaly observations (Figure 3 Right).

#### 3.3.4. Thymus–Body Ratio (TBR)

The results showed a pronounced reduction in TBR in challenged broiler chickens (G1), suggesting thymic atrophy, whereas in control chickens (G2), the TBR increased (Figure 3 Right).

### 3.4. Microscopic Lesions

#### 3.4.1. Bursa of Fabricius

**Figure 4:**
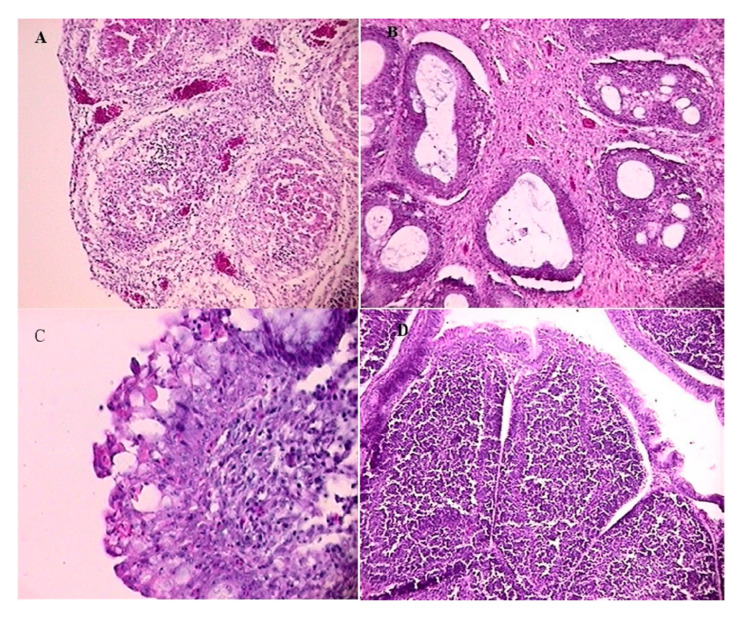
Histopathological changes in the bursa of Fabricius at different dpc in challenged and control broiler chickens. (**A**) At 4 dpc: acute inflammation of the tunica mucosa, with severe heterophil infiltration, lymphoid depletion of follicles, and intrafollicular cysts filled with necrotic debris (lesion score: 3). H&E, x16. (**B**) At 9 dpc: severe fibroplasia of the stroma, extensive lymphoid depletion, and metaplasia of the follicular reticular epithelium. The follicles contain large cysts filled with proteinaceous fluid (lesion score: 4). H&E, x16. (**C**) At 9 dpc: the apex of a plica, showing severe hyperplasia of the lining epithelium and the presence of multiple cysts (lesion score: 5). H&E, x40. (**D**) The control broiler group (G2): normal bursal follicles with no histopathological lesions (lesion score: 0). H&E, x16.

Experiment 1: At 1 dpc, no microscopic lesions were observed. However, an acute inflammation was present at 3 dpc, characterized by an edema of the tunica muscularis and the tunica mucosa, and severe heterophil infiltration in the stroma and follicles, as well as severe lymphoid depletion of the follicles. Many follicles also contained cysts. The inflammation peaked in severity at 4 dpc (Figure 4A), and was associated with vascular congestion in the stroma and a highly undulating epithelium. At 5 dpc, a moderate-to-severe lymphoplasmacytic infiltration was observed in the tunica muscularis. Some follicles had undergone metaplasia of the reticular epithelium, which had turned into pseudostratified epithelium. The lining epithelium was corrugated and hyperplastic. Lymphoplasmacytic infiltration extended to the stroma at 7 dpc, and the tunica muscularis had undergone fibroplasia. Metaplasia of the reticular epithelium was observed in most of the follicles, which were almost completely depleted and contained large cysts. Finally, at 9 dpc, the lesions present at 7 dpc had increased in severity (Figure 4B), and in some chickens, both the tunica muscularis and the stroma had undergone severe fibroplasia. Only limited follicles remained, which had completely lost their architecture. Hyperplasia of the lining epithelium was most severe at the apex of the plicae (Figure 4C), and multiple cysts of varying sizes were found in the lining epithelium. The mean lesion score gradually increased, until it reached 3.75 at 9 dpc (Table 2). No microscopic lesions were observed in the BF of the control broiler group (G2), and the BF follicles appeared perfectly normal, with dense lymphocyte populations (Figure 4D).

**Figure 5:**
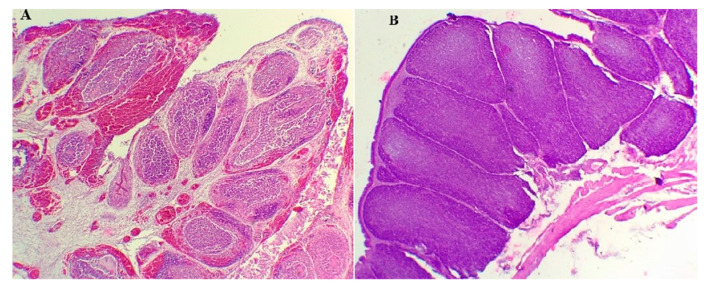
Histopathological changes in the bursa of Fabricius in challenged and non-challenged SPF chickens. (**A**) Challenged SPF chicken (G3), 3 dpc: bursa follicles showing severe hemorrhages, edema, and lymphoid depletion, with follicular necrosis and degeneration, widening of the interstitial space, and architectural disruption (lesion score: 3). H&E, x4. (**B**) Non-challenged SPF chicken (G4): intact bursa follicles with a normal histological structure and no lesions (lesion score: 0). H&E, x4.

Experiment 2: Microscopic lesions revealed the degeneration and necrosis of lymphocytes in the cortex of bursa follicles. Lymphocytes were replaced by heterophils. In many follicles, edema and enlargement in interfollicular areas were observed. Hemorrhages were also present in the cortex and medullary regions (Figure 5A). In some follicles, vacuolization and necrosis in the medullary area were observed. Bursal lesions were significantly more severe in the SPF challenged group (G3); the mean bursal lesion score in dead SPF chickens (G3) (14/15) at 3 and 4 dpc reached a value of 3. In the negative SFP control group (G4), no histological lesions were detected, and the BF follicles showed high lymphocyte populations (Figure 5B).

#### 3.4.2. Spleen

**Figure 6:**
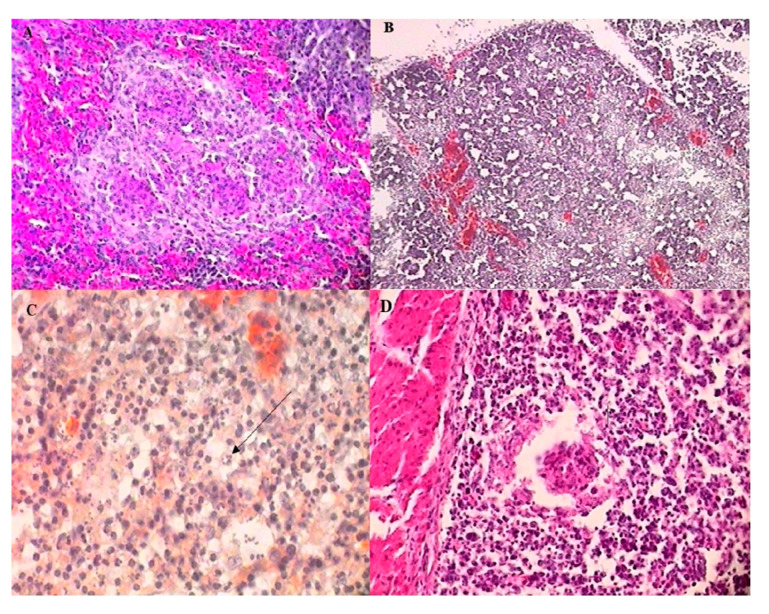
Histopathological lesions in the lymphoid organs of challenged broiler chickens at different dpc. (**A**) Splenic parenchyma (3 dpc): severe hyperplasia of reticular cells and marked hyperemia in the red pulp (lesion score: 2). H&E, x40. (**B**) Thymic cortex (4 dpc): severe lymphoid depletion, with hyperemia and hemorrhages (lesion score: 3). H&E, x16. (**C**) Thymic medulla (4 dpc): extensive pyknosis and karyorrhexis (black arrow), indicating severe cellular degeneration (lesion score: 3). H&E, x100. (**D**) Lamina propria of a caecal tonsil (4 dpc): necrosis of a germinal center, with severe lymphoid depletion in the lamina propria (lesion score: 3). H&E, x40.

Spleen lesions first appeared at 3 dpc and showed severe hyperplasia of the reticular cells, as well as severe red pulp hyperaemia (Figure 6A). At 4 dpc, macrophages accumulated around blood vessels, and from 5 to 7 dpc, periarteriolar lymphoid sheaths were depleted, as well as the germinal centers, seen in smaller numbers. Lymphoid repopulation was observed at 9 dpc, and germinal centers were numerous and reactive. The mean lesion score was between 2 and 2.5 from 3 to 7 dpc, then decreased (Table 2).

#### 3.4.3. Thymus

Severe hyperemia and hemorrhages were observed at 4 dpc. There was a severe lymphoid depletion of the cortex (Figure 6B), necrosis of the medullary zone (Figure 6C), and the corticomedullary junction was not apparent. At 5 dpc, hyperplasia of the reticular cells was observed. Finally, from 7 dpc onwards, only hyperemia and hemorrhages were noted. The mean lesion score for the thymus was 3, reaching its maximum at 4 dpc (Table 2).

#### 3.4.4. Caecal Tonsil

Lesions in the tonsil appeared at 3 dpc, and were characterized by a severe lymphoid depletion of the lamina propria and extensive necrosis of the germinal centers. These centers were reduced to small masses of necrotic debris (Figure 6D). At 5 dpc, several germinal centers were moderately to significantly depleted, but showed no necrosis. The lamina propria was partially or completely repopulated. At 7 dpc, the germinal centers were regenerated, and some contained large, mildly basophilic lymphoid cells. Finally, at 9 dpc, no differences were detected between the control group (G2) and the challenged group (G1). The mean lesion score reached 3 at 4 dpc, and decreased at 5 dpc (Table 2).

### 3.5. Viral Load in Bursa of Fabricius and Viral Excretion in Cloacal Swabs

#### 3.5.1. Bursa of Fabricius

**Figure 7:**
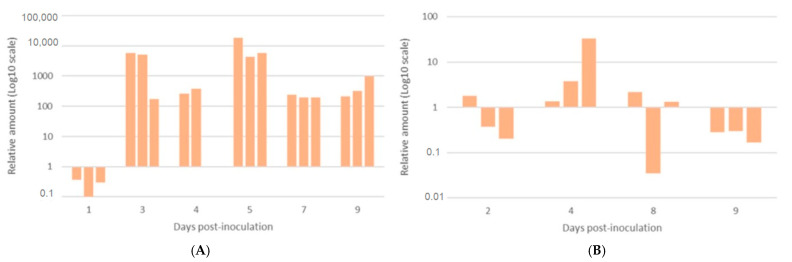
Relative quantification of the vvIBDV viral load in challenged broiler chickens (G1). (**A**) The viral load in the bursa of Fabricius: low at 1 dpc, and increasing to a peak at 3 and 5 dpc (Ct = 11.8), followed by a gradual decline until 9 dpc (Ct = 29.5). (**B**) Viral excretion in cloacal swabs: initially low (Ct = 29.3 at 1 dpc), and reaching a peak at 4 dpc (Ct = 21), then gradually decreasing by 9 dpc (Ct = 28.7).

Viral IBDV detection by qRT-PCR in the BF challenged broiler chickens (G1) revealed that the viral load was low at 1 dpc and increased at 3 dpc, reaching a threshold cycle (Ct) of 13.5. It remained high over the following days of the experiment, peaking (Ct = 11.8) at 3 and 5 dpc (Figure 7A). The viral load gradually decreased towards the end of the experiment, with a minimum Ct value of 29.5 at 9 dpc. In the SPF group (G3), most birds died at 3 and 4 dpc (14/15). The viral load in this group was very high, with Ct values ranging between 12.9 and 18.9. In the survivor SPF bird (G3), virus detection was low at 10 dpc (Ct = 21.8).

#### 3.5.2. Viral Excretion in Cloacal Swabs

In the commercial broiler group (G1), viral excretion was low at 1 dpc (Ct = 29.3), but peaked at 4 dpc (Ct = 21). By day 8 pc, it had decreased significantly (Ct = 25.7), and remained low at 9 dpc (Ct = 28.7) (Figure 7B).

## 4. Discussion

Infectious bursal disease ranks among the most significant viral diseases affecting the poultry industry worldwide. The IBDV exhibits varying degrees of virulence, with vvIBDV strains being particularly responsible for high mortality rates and reduced performance in poultry. Recently, in Morocco, molecular characterization of the full IBDV genome revealed the presence and spread of vvIBDV genotype strains in poultry flocks. These strains contain all genetic markers of virulence, along with specific amino acids substitutions unique to Moroccan IBDV isolates [15]. However, studying the virulence evolution of Moroccan vvIBDV isolate is necessary for forming a correlation between genotype and pathotype, and to better understand the epidemiology, pathogenicity, and prevention of Gumboro disease in Morocco. This study aimed to determine and evaluate the pathogenicity of the recent Moroccan vvIBDV strain 1/chicken/Morocco/IB19/2017 (accession no. MK580160 and MK580164) by evaluating the clinical signs, mortality rate, and both macroscopic and microscopic lesions in various lymphoid organs of commercial broilers (G1) under controlled experimental conditions. The second experiment was intended to reproduce infectious bursal disease in light-breed SPF chickens, in whom mortality rate reach up to 100% [19]. Using SPF chickens is considered the standard model to assess the pathogenicity of IBDV.

The typical clinical signs of acute IBD observed, such as depression, prostration, ruffled feathers, and watery or white diarrhea, were consistent with those previously described by Berg [6] for acute infectious bursal disease. The mortality that occurred at 4 dpc for broilers (G1) reached 10%, which is very low in comparison to SPF chickens, where the mortality appeared at 3 to 5 dpc, and reached 84% at 5 days. Approximatively the same mortality rate was found by Li et al. after three to five dpc with vvIBDV strain isolate in China in 2011 [21]. Therefore, the mortality rate in SPF chickens (G3) is eight times higher than the mortality in broilers (G1); this can be explained by the increased genetic sensibility of light-breed chickens to infection with very virulent IBD viruses compared to heavy breeds, as has been reported in several studies [22]. The mortality rate (10%) in broilers (G1) observed in this study was similar to the rate reported by Eterradousi and Saif and Berg et al. [2,19] for vvIBDV field outbreaks in broilers. These authors reported mortality rates ranging between 5 and 15% and 10 and 25%, respectively.

The gross lesions observed in SPF chickens (G3) in our work were more severe than those in the commercial broilers (G1); in particular, the BF in SPF (G3) chickens appeared very hemorrhagic, resembling black cherries, in most deceased birds (G3). However, the macroscopic lesions in commercial broilers (G1) were characterized by small yellowish bursae [2,6]. The volume of the BF decreased in challenged broilers (G1), while it moderately increased in control chickens (G2), corresponding to normal growth of the BF, as demonstrated by Cazaban et al. [23]. We observed significant regression in the bursa body ratio in challenged broilers (G1), similarly to the observations of Tanimura et al., Hoque et al., and Li et al. [11,16,21] in their studies on the impact of vvIBDV strains on this parameter.

According to Lucio and Hitchner [24], a mean bursa/body index below 0.70 in broilers indicates bursal atrophy, while the mean bursa/body index in our experiment on broilers (G1) was 0.54, indicating severe bursal atrophy. Takahashi et al. reported severe bursal atrophy after challenge with a novel antigenic variant of IBDV in Japan [25].

The spleen–body ratio increased in challenged broilers (G1), indicating splenomegaly at 5, 7, and 9 dpc; this observation was also described by Hoque et al. [16] for the vvIBDV 92/04 strain. The thymus–body ratio decreased significantly in challenged broilers (G1), which could be attributed to thymic atrophy. This observation was also made by Tanimura et al. [11]. However, thymic microscopic lesions of broilers (G1) were transient, and only visible in deceased chickens, contrasting with the severe thymic lesions observed in SPF chickens as described by Inoue et al. and Sharma [1,26].

The histological lesions of the BF in challenged broilers (G1) did not show lymphoid regeneration at 9 dpc, a finding also described by Ignjatovic [27], as is typical of infections by vvIBDV strains of higher virulence. The mean lesion score was 3.75; this is similar to the results obtained by Nakamura et al. [28], who reported a score of 3.3 for the 90–11 Japanese vvIBDV strain, and by Van den Berg et al. [19], who reported a score of 3.8 for 849 VB European vvIBDV strain. Elbestawy and co-authors [29] reported specific histopathological changes associated with the vvIBDV-A3B2 strain isolate in Egypt in 2021. Additionally, the mean lesion scores for the spleen, caecal tonsil, and thymus were similar to those obtained in previous studies of very virulent strains from Japan and the Netherlands [11,30,31]. BF lesions observed in SPF chickens were very pronounced.

Periportal lymphoid infiltrations in the liver have been previously described for infectious bursal disease by Ley et al. [32]. Degeneration of tubular epithelial cells in the kidneys was observed in our study; these lesions were also found in sacrificed chickens after 7 dpc, and could be explained by the vvIBDV pathotype of the Moroccan strain used in this study.

Finally, the viral load in the BF and viral excretion during an IBDV infection has not been extensively studied before. Li and co-authors [33] assessed the viral load in the BF and viral excretion via RT-PCR relative quantification, following experimental infection with the vvIBDV European strain DK01. At 3 dpc, the relative viral load in our study was over three times higher than the value obtained by the same authors when using DK01 as vvIBDV challenged strain. These observations suggest a higher replication of Moroccan IBDV in the BF and the other lymphoid organs, and clearly indicates the high virulence of this Moroccan strain.

The results of the current study showed the high pathogenic potential of the Moroccan IBD virus, which is genetically characterized as very virulent virus based on both genome segments. These results confirm earlier reported observations that SPF light-breed chickens are more susceptible to vvIBDV infection than broilers [22]. However, differences in susceptibility to IBDV infection have been observed based on genetic variations in SPF chicken lines [34]. Nielsen and collaborators reported higher susceptibility to IBDV infection in layer chickens compared to broilers. In contrast with previous studies, our investigation is the first to combine several parameters to describe the pathogenicity of Moroccan very virulent IBDV, particularly by evaluating both viral load excretion and bursal viral load, providing data up to 9 dpc. Additionally, the pathogenicity of Moroccan vvIBDV is increased when both genome segments A and B are found to be very virulent. We demonstrated that this synergy between the A3 segment and B2 segment generates high pathogenicity of the virus. Further studies are needed to evaluate the level of protection conferred by vaccines currently used in the country against the recent Moroccan vvIBDV isolates.

## 5. Conclusions

All the studied criteria in both chicken lines, SPF and broiler chickens—including mortality rate, clinical signs, bursa–body index, macroscopic and microscopic lesions of the affected organs, and a high viral load found in the bursa of Fabricius and in cloacal swabs—characterize the high virulence of Moroccan IBDV strains circulating in Morocco, and corroborate the results of previous studies on the pathogenicity of IBDV.

## Figures and Tables

**Table 1:** Vaccination schedule for broiler chickens.

Chicken Age (Days)	Vaccines
4 days	Prime vaccination: infectious bronchitis disease (live attenuated vaccine; H120) Prime vaccination: Newcastle disease (live attenuated vaccine; VG/GA)
9 days	Booster dose: Newcastle disease (live attenuated vaccine; VG/GA) Prime vaccination: Avian influenza H9N2 (bivalent inactivated vaccine)
16 days	Booster dose: infectious bronchitis disease (live attenuated vaccine; IB88)

**Table 2:** The mean lesion scores of histological sections of lymphoid organs in challenged broiler chickens (G1) from Day 1 to Day 9 post-challenge. Lesion scores are provided for the bursa of Fabricius, spleen, thymus, and caecal tonsil, illustrating the progression of histopathological changes following vvIBDV infection.

Organ	Mean Lesion Scores at Days Post-Challenge					
	1	3	4	5	7	9
Bursa of Fabricius	0/5	2/5	3/5	2.67/5	3.33/5	3.75/5
Spleen	0.33/4	2/4	-	2/4	2.5/4	1.6/4
Thymus	0/3	0/3	3/3	0/3	0.25/3	0/3
Caecal tonsil	0/3	0/3	3/3	2/3	0/3	0/3

## Data Availability

The data supporting the results of this study are the same as those presented in the preprint version of the manuscript, which is available at https://orcid.org/0000-0001-8301-7243 (accessed on 24 March 2025). No new data were generated or analyzed in addition to those already provided in the preprint.

## Abbreviations

The following abbreviations are used in this manuscript:

BBI = bursa–body index; BBR = bursa–body ratio; BF = bursa of Fabricius; CT = cycle threshold; dpc = days post-challenge; dsRNA = double-stranded RNA; EID_50_/_mL_ = 50% embryo infective dose per milliliter; G1 = challenged broiler group; G2 = broiler control group; G3 = challenged SPF group; IBDV = infectious bursal disease virus; IB19 = IBDV challenge strain; Kb = kilobase; kDa = kilodalton; ORF = Open Reader Frame; pp = polyprotein; SPF = specific-pathogen-free; SBR = spleen–body ratio TBR = thymus–body ratio; vv = very virulent.
